# Muscle Coactivation during Stability Exercises in Rhythmic Gymnastics: A Two-Case Study

**DOI:** 10.1155/2018/8260402

**Published:** 2018-04-01

**Authors:** Alicja Rutkowska-Kucharska, Agnieszka Szpala, Sebastian Jaroszczuk, Małgorzata Sobera

**Affiliations:** ^1^Department of Biomechanics, University of Physical Education in Wroclaw, Wroclaw, Poland; ^2^Laboratory of Biomechanical Analysis, University of Physical Education in Wroclaw, Wroclaw, Poland; ^3^Department of Athletics and Gymnastics, University of Physical Education in Wroclaw, Wroclaw, Poland

## Abstract

Balance exercises in rhythmic gymnastics are performed on tiptoes, which causes overload of foot joints. This study aimed to evaluate the engagement of muscles stabilizing ankle and knee joints in balance exercises and determine exercises which may lead to ankle and knee joint injuries. It was hypothesized that long-term training has an influence on balance control and efficient use of muscles in their stabilizing function. Two rhythmic gymnasts (8 and 21 years old) performed balances on tiptoes (side split with hand support, ring with hand support) and on a flat foot (back split without hand support exercise). Surface electromyography, ground reaction forces, and kinematic parameters of movement were measured. The measuring systems applied were synchronized with the BTS SMART system. The results show the necessity to limit balance exercises on tiptoes in children because gastrocnemius medialis (GM) and gastrocnemius lateralis (GL) activity significantly exceeds their activity. Ankle joint stabilizing activity of GM and GL muscles in the younger gymnast was more important than in the older one. Performing this exercise, the younger gymnast distributed load on the anterior side of the foot while the older one did so on its posterior. Gymnastics coaches should be advised to exclude ring with hand support exercise from the training of young gymnasts.

## 1. Introduction

Both rhythmic and artistic gymnastics are sports which allow early specialization, and girls as young as 4 years are engaged in regular training. Frequent injuries observed in young children result from an ongoing process of osteosis, which occurs throughout childhood [1, 2]. The injuries often occur during one-foot landing in jumping and one-foot balance exercises [3]. Many balance exercises in rhythmic gymnastics are performed on tiptoes, which causes overload of foot joints [4]. Daily, repetitive tiptoe exercises can be a source of ankle sprains and consequently lead to chronic sports-related injuries in children and adolescents [5, 6]. Injuries to the ankle and foot were most commonly reported in elite and subelite female gymnasts [7, 8]. Conversely, researchers indicate a positive impact of balance exercises on gymnasts' motor system and ability to control their body posture [9]. Gruodyte-Raciene et al. [10] reported that early (from 4 years old) exposure to low-level gymnastic participation confers benefits related to geometric and bone architecture properties during childhood and may improve bone health in adolescence and adulthood. Aydin et al. [11] suggested that gymnastic training has a positive influence on sense of position of the ankle joint and on balance, in addition to increasing muscle tone.

Nearly all muscles stabilizing motor system joints are activated in maintaining balance with one-leg support on tiptoes [12]. Anatomy shows that muscles surrounding joints are both agonistic and antagonistic towards each other. In the literature, they are described based on an analysis of muscle cocontraction and coactivation [13]. Muscle cocontraction is a simultaneous contraction of antagonistic muscle pairs, while muscle coactivation is concurrent activity of muscles surrounding joints. Cocontractions are frequently used to assess muscle engagement in dynamic activities [14, 15], while muscle coactivation in static activities proves its function in maintaining joint stability [16–18].

Maintaining balance on a limited support surface in exercises such as balance on tiptoes in rhythmic gymnastics requires correct knee and ankle joint stabilization. Moreover, muscle stabilization prevents injuries within these muscles [19]. This is understandable considering that ankle and knee joints are submitted to the greatest loads in the human motor system [20, 21]. The quadriceps femoris (QF) and biceps femoris (BF) play a key part in the knee joint stabilization process. The BF is as an important contributor to restraint of anterior tibiofemoral displacement and lateral rotation of the femur relative to the tibia, since both processes have been implicated in anterior cruciate ligament injury [22]. Varied structure of the QF may indicate a diversified activation strategy of motor units within its actions [23]. The tibialis anterior (TA) and gastrocnemius (GAS) are key muscles in the process of ankle joint stabilization [24]. The TA is responsible for dorsiflexion, while the GAS is the strongest muscle-performing plantar flexion. The GAS is involved in pressing the foot to the ground, enables lifting the body on tiptoes, and detaches the heel from the ground such as in walking, running, or jumping [25]. The ankle joint is made of up a combination of bones, whose segments create a bone lever used in maintaining balance. One force applied to the ankle joint produces muscle torque, and the other one produces moment of gravity. In tiptoeing, the distance between the rotation axes of the talocrural joint to the hallux is at least fivefold longer than the distance to the heel [26]. This implies that muscle torque generated by the GAS works against the lever in the least favorable position in a 5 : 1 ratio. Therefore, if a 25 kg gymnast performs one-foot tiptoeing, the GAS and soleus generate a balance force of approximately 1200 N. Coactivation of calf muscles and plantar foot flexors is needed to minimize movements in the ankle joint when performing tiptoe exercise on one foot. Russell et al. [19] considered the ankle joint to be the most important in balance exercises executed on the toes due to its lower limb stabilization. The GAS injuries impede walking since the strength of the remaining flexors is not sufficient to lift the body to the toes [27]. Injury to this muscle is particularly troublesome for gymnasts and ballet dancers [28].

Elements of rhythmic gymnastics' technique, including balance exercises, consist of points and a technique described in the FIG (Fédération Internationale de Gymnastique) Technical Regulations [29]. All age-group gymnasts perform the same elements of the technique. Many elements are executed on flat foot and others on tiptoes. Moreover, it is a sport in which gymnasts manipulate different apparatus (ball, rope, clubs, hoop, and ribbon) which additionally hinder balance. It indicates that a gymnast can perform an exercise with an apparatus only when, according to a coach, their technique is correct. Despite the coach's positive evaluation, application of precise assessment tools may reveal errors leading to foot overload. This study aimed to evaluate an engagement of muscles in stabilizing function of ankle and knee joints in balance exercises in rhythmic gymnastics. Analysis of muscle coactivation in stabilizing function will help to determine exercises which may lead to ankle and knee joint injuries.

It was hypothesized that long-term training has an influence on balance control and efficient use of muscles in their stabilizing function.

## 2. Material and Methods

**Figure 1:**
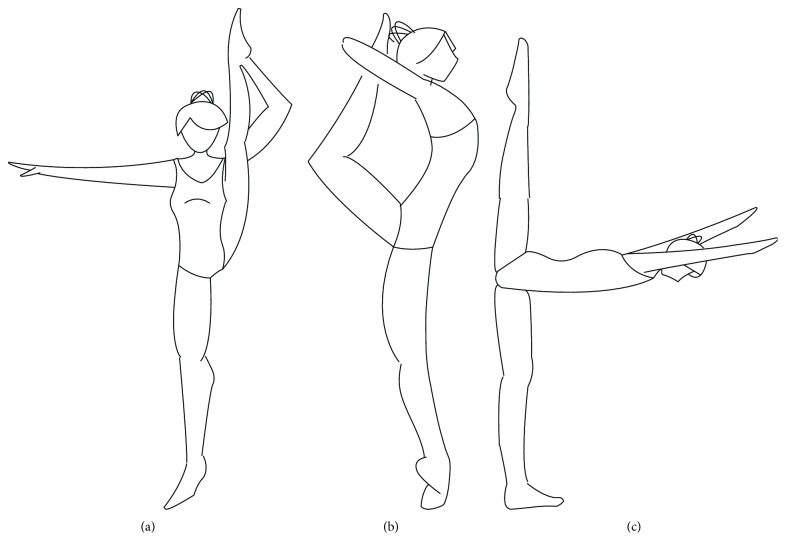
Balances performed by the gymnasts: (a) side split with hand support (SSpS), (b) ring with hand support (RS), and (c) back split without hand support (BSp).

Two rhythmic gymnasts participated in the study: the younger subject (8 years old, body height = 1.35 m, body mass = 29 kg, body mass index (BMI) = 16 kg/m^2^) with 4 years of experience (competitor A) at a third class level and an older one (21 years old, body height = 1.67 m, body mass = 61.5 kg, BMI = 22 kg/m^2^) with 14 years of experience (competitor B) at National Masterclass level. The competitors performed, on preferred leg, in the same order, three repetitions of balances on tiptoes (side split with hand support—SSpS, ring with hand support—RS) and on a flat foot (back split without hand support—BSp) (Figure 1).

The participants were informed about the aims and methodology used in the experiment. The experiment was approved by the local ethics committee and conducted in accordance with the Declaration of Helsinki.

### 2.1. Measuring Procedure

**Figure 2:**
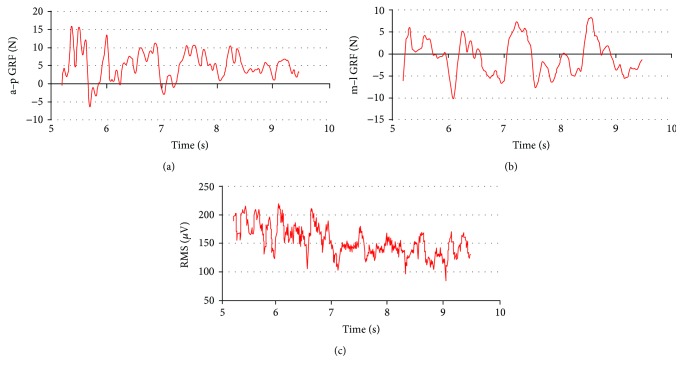
Typical course of a–p GRF (a), m–l GRF (b), and EMG signal (c) support leg during flat foot standing in back split without hand support. Positive values represent components of the posterior and lateral GRF; negative values are components of the anterior and medial GRF.

Surface electromyography (EMG), ground reaction forces (GRF), and kinematic parameters of movement were measured. The measuring systems applied were synchronized with the BTS SMART system. Examples of synchronized signals for the analyzed exercise phase are presented in Figure 2. The study was carried out at the Laboratory of Biomechanical Analysis, which is certified by the ISO quality certificate number 1374-b/3/2009, PNEN ISO9001:2009.

#### 2.1.1. EMG

The EMG signal was recorded for 14 knee and ankle joint flexors and extensors of the lower limb performing the balances. The recorded muscles were knee extensors (rectus femoris—RF, vastus lateralis—VL, and vastus medialis—VM), knee flexors (BF), ankle joint extensors (TA), and ankle joint flexors (gastrocnemius lateralis—GL, gastrocnemius medialis—GM).

Disposable surface electrodes were fixed to the skin in bipolar configuration above the muscle belly, along the muscle fibers. Twenty-eight active electrodes (46 × 22.5 mm) with a solid gel (Ag-AgCl, No R–LFR-310, Bio-Lead-Lok, Poland) were attached to the muscles and 1 reference electrode to the fibula head of the right lower limb. The electrodes were spaced at 20 mm intervals.

Measurement was performed following the recommendations of the SENIAM project (surface electromyography for the noninvasive assessment of muscles) [30]. The place of electrode-skin contact was cleaned and degreased. The electrodes were fixed with respect to an individual course of muscle fibers.

Muscle activity measurements in maximum voluntary contractions were used to normalize EMG signals. Upon a signal, the subject performed a maximum voluntary isometric contraction (MVIC), used as a reference point in dynamic analyses [31].

MVIC measurements for 12 muscles were taken in a sitting position in a multifunction armchair (UPR - 01 A/S, SUMER, Opole, Poland). The angle at hip and knee joints was 90° and at the knee joint 75° (for MVIC measurements of the knee extensors) and 30° (for MVIC measurements of the knee flexors). Full knee extension was 0 degree [32].

In MVIC measurements of knee flexors and extensors, the resistance part of the measuring stand was placed in the ankle joint at the front and back of a shank. MVIC measurements for ankle flexors (GL and GM) were taken when the resistance part of the stand was positioned in the metatarsophalangeal joints area, at the plantar surface of the foot. MVIC measurements for TA muscles (right and left) were taken in a standing position. While performing MVIC, a subject resisted an assistant's hand placed at the dorsal foot.

The measurements were taken with a TeleMyo 2400T G2 (Noraxon Inc., USA) transmitter with a frequency from 10 Hz to 1500 Hz.

A PC with BTS SMART system with a sampling frequency of 1 kHz was used to analyze the EMG signal. The raw EMG signal was processed with the Butterworth filter with a low-pass and high-pass frequency of 300 Hz and 10 Hz, respectively. Next, the EMG signal module was calculated to estimate the root mean square (RMS) amplitude using a 0.1 s window. The maximum amplitude was used as a reference point (100%) for muscle activity recorded in static trials.

#### 2.1.2. GRF

A gymnast stood on Kistler 9286AA-A plate (at the frequency of 1 kHz and size of 600 × 400 mm) and performed balance exercises. A anterior-posterior (a–p GRF) and medial-lateral (m–l GRF) components of GRF were recorded. Actual data have been normalized to the gymnasts' body weight (BW) and expressed in relative values. Values calculated for the horizontal GRF are as follows: anterior GRF (aF), posterior GRF (pF), medial GRF (mF), and lateral GRF (lF).

Body balance of the subjects was also examined with the Kistler plate. Calculations were based on a cut-off record of the course of the center of pressure (COP) in time. The course of the COP (COP time series) was used to calculate body sway indices:

- COP amplitude in medial-lateral (COPX) and in anterior-posterior direction (COPY)
- COP amplitude in anterior-posterior direction (COPY)

COP amplitude means the distance between two extreme COP shifts on the surface of a foot support in both directions medial-lateral and anterior-posterior. COP amplitude represents body sways in the frontal and sagittal planes.

Values of COP amplitude for both gymnasts, characterized by different body measurements, were normalized to allow the comparison of the results obtained. The normalization was in medial-lateral direction and transverse foot measurements and anterior-posterior direction and longitudinal foot measurements taken from a foot print of a supporting limb. Longitudinal foot measurement is the length between the heel and the hallux, while transverse foot measurement is the distance between the contour of the first and fifth metatarsal head. The precision of measurements was 1 mm.

#### 2.1.3. Movement Analysis

Body movements of the gymnasts were analyzed with the three-dimensional BTS SMART system. The BTS SMART system comprised 6 calibrated infrared video cameras at the frequency of 120 Hz and resolution of 640 × 480 px.

The gymnasts had a set of reflective markers attached to their skin at the following anatomical points: spinous processes of the seventh cervical vertebra, left and right acromion, anterior superior iliac spine (left and right), center of the sacrum at the height of the anterior superior iliac spine, greater trochanter of the femur (right and left), lateral epicondyle of the femur (right and left), head of the fibula (caput fibulae, right and left), lateral ankle (lateral malleolus, right and left), head of the fifth external metatarsal bone (right and left), and tuber calcanei of the right and left limb and markers placed on rods: at the thigh coplanar with the lateral epicondyle of the femur and greater trochanter (right and left) and at the shin coplanar with head of the fibula and lateral malleolus (right and left). Phase of a correctly performed exercise for the selected static activities was verified with a stick diagram.

### 2.2. Statistical Analysis

Statistical analysis was carried out based on data from the BTS system. The analyzed fragments of EMG and GRF signals lasted for 3 s. In that time, a gymnast performed a task in compliance with judging requirements.

An Excel spreadsheet was used to organize data. Statistical analysis was carried out with the Statistica 12.0 software package (StatSoft Inc., Tulsa, Oklahoma, USA). The significance of differences of mean values obtained by the younger (A) and older (B) gymnast was examined with Student's *t*-test for independent variables. Differences in muscles' activity in each exercise and between exercises for each gymnast were verified with repeated measures analysis of variance (ANOVA) and post hoc analyses were performed with LSD test (least significance difference test).

The results of the statistical analysis include *t*—the value of the *t*-test, *F*—the value of *F* test, *p*—the probability level for the tests, and df—number of degrees of freedom. The level of significance was set at *p* < 0.05.

## 3. Results

### 3.1. Agonist-Antagonist Activity during Stabilization of Ankle and Knee Joint

Coactivity of agonist and antagonist muscles in joint stabilization was compared (i) in balance exercises, (ii) between the muscles activity in ankle and knee joint, and (iii) between the subjects (Table 1). Relative values were analyzed and presented as the percentage.

#### 3.1.1. Coactivity of Muscles in Balance Exercises

The statistical analysis (ANOVA) revealed only for one muscle—BF, significant differences (df = 2, *F* = 6.58, and *p* = 0.02) in the activity between balance exercises in both subjects. Post hoc analysis indicated the significance of differences in the younger gymnast between SSpS and BSp exercise and between RS and BSp, and the older gymnast between the RS and BSp and RS and SSpS exercises.

#### 3.1.2. Coactivity of Muscles in Joint Stabilization

In the process of stabilization of the ankle joint, statistical analysis revealed significant differences between muscle activity (TA, GL, and GM) in both gymnasts in the SSpS exercise (df = 2, *F* = 6.02, and *p* = 0.02) and RS (df = 2, *F* = 35.17, and *p* = 0.0001). There were no significant differences for the BSp exercise (df = 2, *F* = 2.49, and *p* = 0.14). Interestingly, GL and GM muscles presented significantly higher activity in relation to TA in the SSpS and RS exercise in the younger gymnast and in the older one only in the RS exercise. An important difference between GL and GM was demonstrated in SSpS and RS only for the younger gymnast.

In the process of stabilizing of the knee joint, significant differences in muscle activity (BF, FR, VM, and VL) were found in the SSpS exercise (df = 3, *F* = 22.48, and *p* = 0.0001) and RS (df = 3, *F* = 10.95, and *p* = 0.0009) for both competitors. There was no significant difference in muscle activity for the BSp exercise (df = 3, *F* = 2.02, and *p* = 0.16). The BF muscle showed significantly higher activity in relation to the knee extensors in the younger gymnast in the SSpS and RS exercises. On the other hand, in the older gymnast, the BF activity was significantly lower in the SSpS exercise and significantly higher in the RS compared to the knee extensors.

#### 3.1.3. Coactivity of Muscles between Subjects

The comparison of differences in muscles' activity based on statistical analysis revealed the following findings:

- SSpS—statistically significantly higher EMG activity of GM (*t* = 3.91; *p* = 0.017) and BF (*t* = 5.81; *p* = 0.004) in the younger gymnast and VM (*t* = −2.85; *p* = 0.046) in the older one
- RS—statistically significantly higher EMG activity of GM (*t* = 15.69; *p* = 0.000), GL (*t* = 6.88; *p* = 0.002), and TA (*t* = 9.09; *p* = 0.000) in the younger gymnast and VM (*t* = −16.01; *p* = 0.000) and VL (*t* = −14.22; *p* = 0.000) in the older one
- BSp—statistically significantly higher EMG activity of TA (*t* = 15.94; *p* = 0.000) and GM (*t* = 6.92; *p* = 0.002) in the younger gymnast (Table 1)

### 3.2. Foot Load Evaluation

Values of the horizontal GRF indicated foot load in SSpS and RS performed on tiptoes.

The younger gymnast put load on the medial foot in BSp while the older one (B) did so in RS. BSp was performed on a flat foot. Performing this exercise, the younger gymnast (A) distributed load on the anterior side of the foot while the older one (B) did so on its posterior. Statistical analysis of this exercise showed significant differences between the two gymnasts in the anterior GRF (aF) (*t* = 3.13; *p* = 0.035) and the posterior GRF (pF) (*t* = −4.43; *p* = 0.011).

Statistically significant differences in force values pF were observed only for the older gymnast between SSpS and RS (*t* = −4.34; *p* = 0.049) and between SSpS and BSp (*t* = −4.32; *p* = 0.049). A single significant difference in the mF was found between RS and BSp (*t* = 4.8; *p* = 0.044) (Table 2).

### 3.3. Balance Evaluation

COP shifts were applied to compare quality of posture control in balance exercises in both gymnasts (A and B) (Table 3).

The values obtained were normalized in order to allow comparison of balance maintained by the gymnasts of different height and limb length. Comparison of the normalized values of COP amplitude in the lateral and anterior-posterior directions of gymnast A and B in all balance exercises showed that the percentage of width and length of the supporting foot used in the exercise was much lower in gymnast B (Table 3).

As stated in the regulations of the FIG, a rhythmic gymnast needs to acquire skills to perform choreography at a certain difficulty level [29]. Balance exercises are part of a general division of difficulty components into groups. The evaluation criterion for this exercise is maintaining the position for 1 second, as specified in the regulations (Figure 1). The tested gymnasts complied with the criteria for all the exercises. However, such evaluation was not sufficient to assess muscles' cooperation in stabilizing function. Therefore, the researchers applied an accurate method of evaluating balance based on COP shifts [33, 34]. COP amplitude shifts in both anterior-posterior and medial-lateral directions were used to evaluate the process of maintaining balance. Higher normalized parameters of COP amplitude of body sways showed that the younger gymnast (A) revealed poorer balance control in comparison to the older one (B). Higher amplitude of COP shifts in the younger competitor in RS exercise showed that maintaining balance in this exercise was arduous for her.

## 4. Discussion

Analysis of the engagement and interaction of the agonistic and antagonistic muscles in the stabilizing function of the ankle joint in balance exercises provided data on those activities which exerted the greatest load on competitors' motor system. Our study was deliberately based on tiptoe and flat foot exercises. In tiptoe balance exercises, the selected exercises were SSpS and RS. Our findings confirmed the major function of the GAS in ankle joint stabilization, particularly in the younger competitor. The said muscles were highly activated in the RS exercise—the activity of GM and GL constituted 200% of their regular activity in MVIC. In addition, the range of medial and lateral GRF values was the widest in this exercise. In the 3-second balance exercise, the COG shifted significantly from the medial towards the lateral direction. If muscles' activity significantly exceeds that measured in MVIC, muscle fatigue prevents their stabilizing function (source), which may result in spraining the talocrural joint. Excessive GM activity was observed in the younger gymnast. Activity of GM in the older gymnast was invariably higher than GL, which indicated medial foot loading. Higher medial GRF (mF) values observed in all exercises performed by the younger gymnast and in tiptoe exercises executed by the older one also indicated such loading. This proved that the first metatarsophalangeal joint is prone to overload. Comparison of the EMG results obtained in our research with those of other authors testing muscles' coactivation in tiptoe exercises is challenging, since they do not follow the recommended procedure and do not normalize EMG signals to the MVIC. Saito and coauthors [35] evaluated EMG amplitude of TA and RF for a single and repetitive tiptoe movement to investigate the influence of repetition on anticipatory postural adjustments between these tasks. They presented the results obtained as absolute values of millivolts and observed higher muscle activity in the single task in comparison to the repetitive task (TA: 0.72 versus 0.68 mV; RF: 0.68 versus 0.62 mV). Tanabe et al. [17] analyzed GL, GM, and TA activity in tiptoe standing and quiet standing. The results showed that the EMG amplitude of the tested muscles expressed in microvolts was higher in tiptoe standing in comparison to quiet standing (GM: 99.5 versus 12.9 *μ*V; GL: 36.9 versus 6.9 *μ*V; TA: 21.2 versus 9.8 *μ*V). The scientific literature also presents analyses of muscles' coactivity in a lower limb in grand plié and demi plié in ballet and modern dancers. However, there are differences in the movement structure of these exercises in comparison to tiptoe movement [36, 37].

Agonist and antagonist activity in the knee joint stabilization is influenced by characteristics of a performed exercise and related to the anterior-posterior foot load. a–p GRF values confirmed SSpS to be this type of exercise. The highest agonist and antagonist values in this exercise were obtained by the youngest gymnast. Interestingly, BF participation was greater than knee joint extensors' participation. Its highest activity was observed in the RS exercise performed by the younger competitor. Such correlation between the agonist and antagonist muscles was not found in the older competitor. It is worth noting that the group of knee joint flexors of the support limb uses only approximately 60% of their strength capacity at their maximum strength [38]. No unequivocal correlations between coactivation of knee flexors were confirmed. Statistically significant activity of VM in comparison to VL and VM to RF was only observed in exercises performed by the older gymnast.

Rhythmic gymnastics is a sport in which girls at the age of 8 perform many balances executed on the toes. Due to that, they score more points. However, they are able to do it only after a few years of daily training. Ankle joint stabilization in these exercises is provided by the cooperation of agonistic and antagonistic muscles. There have been no previous publications tackling the problem of ankle joint stabilization in balance exercises performed by rhythmic gymnasts. It has been discussed in studies on ballet dancers, since they are also prone to ankle joint injuries. Ritter and More [20] reported that ballet dancers performing maximum plantar flexion in balance position on point are liable to lateral ankle sprains and sequelae such as inflammation of tendons, especially peroneals, which lead to chronic ankle instability. The authors proved a key role of peroneal muscles in stabilizing the ankle joint and that their overuse may provoke tendonitis. Tiptoe exercise performed by rhythmic gymnasts differs from that performed by ballet dancers. Nevertheless, they still need to perform maximum plantar flexion and maintain one-leg balance on the forefoot requiring tension of the gastrocnemius. These findings are confirmed by different researchers who stated that the Achilles tendon is highly prone to overload in both rhythmic sportive and artistic gymnasts [28].

## 5. Conclusion

Results of research in competitive sport aim to be applied in practice. In early specialization, sports such as rhythmic and artistic gymnastics elements of a technique should be adapted to the child's motor abilities. The obtained results show the necessity to limit balance exercises on tiptoes in children because GM and GL activity significantly exceeds their activity. Gymnastics coaches should be advised to exclude RS exercise from the training of young gymnasts.

## Figures and Tables

**Table 1:** Mean values and standard deviation of the EMG amplitude normalized to MVIC (%) of the support leg muscles of gymnasts A and B in balance exercises. Statistically significant differences between means: letters “a–i” in the upper index.

	Muscle	Gymnast A			Gymnast B		
		Side split with hand support (SSps)	Ring with hand support (RS)	Back split without hand support (BSp)	Side split with hand support (SSps)	Ring with hand support (RS)	Back split without hand support (BSp)
Ankle joint	Antagonist						
	TA	74.96 ± 17.89	53.45 ± 6.23	43.59 ± 2.72	48.52 ± 3.66	19.25 ± 1.89^c^	17.68 ± 0.71^c^
	Agonist						
	GM	135.39 ± 31.32^a^	187.16 ± 12.85^a^	152.59 ± 24.75	64.45 ± 1.92^c^	68.89 ± 2.34^a,c^	53.69 ± 0.67^c^
	GL	123.46 ± 47.92^a,d^	214.18 ± 39.63^a,d^	91.91 ± 76.26	47.64 ± 6.11	56.36 ± 2.14^a,c^	27.44 ± 2.74
Knee joint	Antagonist						
	BF	65.62 ± 16.35	45.69 ± 14.49	17.13 ± 15.93^g,h^	10.62 ± 1.43^c^	36.34 ± 6.97^i^	8.98 ± 1.73^h^
	Agonist						
	VM	30.73 ± 5.33^b^	5.15 ± 1.06^b^	9.56 ± 8.02	51.13 ± 11.18^b,c^	34.91 ± 3.04^c^	21.22 ± 0.19
	VL	27.55 ± 4.14^b^	3.37 ± .032^b^	8.56 ± 8.88	30.72 ± 8.13^b,f^	17.32 ± 1.66^b,c,f^	14.74 ± 1.12
	RF	17.28 ± 6.68^b^	1.83 ± 0.48^b^	4.75 ± 4.68	29.21 ± 13.43^b,e^	10.83 ± 2.79^b,e^	10.27 ± 0.37

^a^Statistically significant difference as compared with TA. ^b^Statistically significant difference as compared with BF. ^c^Statistically significant between gymnast A and B. ^d^Statistically significant between GM and GL. ^e^Statistically significant between VM and RF. ^f^Statistically significant between VM and VL. ^g^Statistically significant between BSp and SSpS. ^h^Statistically significant between BSp and RS. ^i^Statistically significant between RS and SSpS.

**Table 2:** Mean relative values and standard deviation of the anterior (aF) and posterior (pF) GRF and medial (mF) and lateral (lF) GRF of gymnast A and B in balance exercises.

GRF	Gymnast A			Gymnast B		
	Side split with hand support	Ring with hand support	Back split without hand support	Side split with hand support	Ring with hand support	Back split without hand support
aF (% BW)	1.15 ± 0.76	0.64 ± 0.76	1.06 ± 0.46	0.55 ± 0.21	0.48 ± 0.21	0.23 ± 0.04^c^
pF (% BW)	1.02 ± 0.46	1.09 ± 0.67	0.86 ± 0.26	0.71 ± 0.17	2.39 ± 0.83^a^	1.82 ± 0.27^a,c^
mF (% BW)	1.85 ± 1.43	1.18 ± 0.26	1.02 ± 0.71	0.79 ± 0.26	1.37 ± 0.15	0.66 ± 0.21^b^
lF (% BW)	0.39 ± 0.34	1.15 ± 0.70	0.89 ± 0.36	0.72 ± 0.28	0.54 ± 0.30	0.79 ± 0.17

^a^Statistically significant difference as compared with the SSpS exercise. ^b^Statistically significant difference as compared with the RS exercise. ^c^Statistically significant between gymnasts A and B.

**Table 3:** Normal distribution of the COP values of the younger (A) and older (B) gymnast in balance exercises.

	Gymnast A	Gymnast B
*Side split with hand support*		
COPX amplitude	44% of foot width	35% of foot width
COPY amplitude	11% of foot length	11% of foot length
*Ring with hand support*		
COPX amplitude	61% of foot width	41% of foot width
COPY amplitude	16% of foot length	8% of foot length
*Back split without hand support*		
COPX amplitude	57% of foot width	47% of foot width
COPY amplitude	50% of foot length	20% of foot length
